# Telehealth as a Model for Providing Behaviour Analytic Interventions to Individuals with Autism Spectrum Disorder: A Systematic Review

**DOI:** 10.1007/s10803-018-3724-5

**Published:** 2018-08-28

**Authors:** Jenny Ferguson, Emma A. Craig, Katerina Dounavi

**Affiliations:** School of Social Sciences, Education and Social Work, Queen’s University Belfast, 69-71 University Street, Belfast, Northern Ireland BT7 1HL UK

**Keywords:** Applied behaviour analysis, Telehealth, Autism spectrum disorder

## Abstract

Interventions based on applied behaviour analysis are considered evidence based practice for autism spectrum disorders. Due to the shortage of highly qualified professionals required for their delivery, innovative models should be explored, such as telehealth. Telehealth utilises technology for remote training and supervision. The purpose of our study was to systematically review the literature researching telehealth and ABA. We analysed intervention characteristics, outcomes and research quality in 28 studies and identified gaps. Intervention characteristics were: (1) research design (2) participants (3) technology (4) dependent variables (5) aims. Outcomes were favourable with all studies reporting improvements in at least one variable. Quality ratings were significantly low. Implications for future research and practice are discussed in light of identified methodological downfalls.

Autism spectrum disorder (ASD) is a neuro-developmental disorder categorised by impairments in social-communication and restrictions in behaviours or thought patterns (American Psychiatric Association 2013). Recent estimates have suggested 1 in every 59 individuals now have a diagnosis of ASD (Centres for Disease Control and Prevention 2018). The high prevalence and lifelong nature of the condition mean a large demand on services to support individuals spanning across health, social care and education sectors. In the UK, the cost of these services have been estimated at £1.5 ($2.2) million across a lifetime (Buescher et al. 2014), making ASD the costliest medical condition to support (Knapp et al. 2009). More importantly, when compared to neuro-typical individuals, individuals with ASD have been shown to score considerably lower on indicators of life-quality and are less likely to be in paid employment, have rewarding social lives or achieve full independence from their families (Howlin et al. 2004). This highlights the significance of providing successful yet cost effective interventions, which should be based solely upon sound empirical evidence and guided by current evidence based practice (EBP).

Research has indicated that best practice in autism interventions should involve strategies based upon the principles of applied behaviour analysis (ABA) (Makrygianni and Reed 2010; Reichow 2012; Reichow et al. 2018). ABA is an applied science aiming to determine environmental variables that shape socially significant behaviour and design interventions accordingly. Teaching strategies derived from these principles have been identified as EBP (e.g., early intensive behavioural intervention, discrete trial training, functional communication training, functional behavioural assessment, extinction, naturalistic intervention, pivotal response training and task analysis) (Wong et al. 2014).

However, the vast majority of European governments do not fund ABA-based provision; estimates suggest only a third of children with ASD currently access behavioural interventions across Europe (Salomone et al. 2014). In the UK, where an ‘eclectic’ and often ill-defined selection of services is available, access to funded ABA can often be either a postcode lottery or a testament of parental willingness to fight for services at tribunal (Dillenburger 2011; Keenan et al. 2015).

Another barrier to successful ABA treatment is a lack of appropriately trained professionals. To qualify as a Board Certified Behaviour Analyst (BCBA©) individuals must complete Master’s level training in behaviour analysis, undertake an extensive period of supervised practice and pass an exam (BACB 2014). Working under an appropriately qualified supervisor has been shown to correlate with implementation fidelity, therapist job satisfaction and positive child outcomes (Plantiveau et al. 2018; Whiteford et al. 2012; Eikeseth et al. 2008). Difficulties in accessing this expertise is very problematic. In the UK there are currently 314 BCBAs (BACB 2018); considering the number of individuals diagnosed with ASD is estimated to be 695,000 (National Autistic Society 2017), the ratio of 1 BCBA per 2213 individuals with ASD is worryingly small. These shortages are magnified in remote areas of the country, where the lack of local services results in parents travelling long distances to avail of the expertise of qualified professionals. Parents of children with ASD living in rural areas have shown lower levels of service satisfaction and greater difficulties in accessing professional expertise when compared with their urban counterparts (Bulgren 2002; Murphy and Ruble 2012), highlighting the need to update the existing service dissemination model. Whilst it is clear that ABA-based services present best outcomes for individuals with ASD, it is not clear how best to resolve the gap between needs and service access. Alternative models of intervention and training should be explored which may extend the reach beyond a traditional face-to-face model; telehealth has the potential to do this.

Telehealth is the use of communication technology to assist in education and treatment of health related conditions. The availability of internet connections has been growing exponentially in recent years. Current estimates suggest that 88% of all people in the UK accessed the internet at some point in the last 3 months (Office of National Statistics 2016). Researchers have capitalised on these advancements and demonstrated their usefulness for the delivery of health related interventions. These interventions utilise technology to provide remote communication, advice and training using tele-communications software and technology based training platforms. The application of telehealth has been investigated across numerous conditions, such as haemophilia, diabetes, heart disease and depression (Kessler et al. 2009; Webb et al. 2010), proving to be a promising advancement in healthcare government initiatives across the UK (Department of Health 2011; Department of Health, Social Services and Public Safety, Department of Health Northern Ireland 2011; Scottish Government 2011).

An initial examination of the literature indicated that there has been an emergence of a body of research investigating the use of telehealth to provide behaviour analytic provisions to individuals with ASD and the initial findings appear promising; telehealth was shown to reduce costs associated with providing behaviour analytic interventions by up to half (Horn et al. 2016; Lindgren et al. 2015) and was viewed favourably by parents living in rural communities (Salomone et al. 2017). However, a more extensive review capable of identifying the scope, effectiveness and limitations of using telehealth was required. To date there have been five published reviews summarising the body of literature in this area, these reviews have either been too broad (Boisvert et al. 2010; Knutsen et al. 2016) or too narrow (Meadan and Daczewitz 2015; Neely et al. 2016; Parsons et al. 2017). The subsequent paragraphs will discuss the limitations of these reviews in more detail identifying why a specific systematic review in this area in warranted.

Current reviews have not focused specifically on ABA-based interventions and have instead involved overarching reviews including research from wider fields, such as, education, occupational therapy or speech and language therapy. Boisvert et al. (2010) included five ABA-based studies in an overarching review of telehealth based support for individuals with ASD and more recently Knutsen et al. (2016) identified 17 studies that utilised ABA-based interventions amongst a broader review of literature. The selected research investigated Functional Analysis (FA) and associated Functional Communication Training (FCT) (e.g., Barretto et al. 2006; Wacker et al. 2013a, b) or naturalistic teaching strategies (e.g. Vismara et al 2009, 2012, 2013). Outcomes varied within and between studies and individual differences were apparent in interventionist implementation fidelity and child outcomes (e.g. Meadan et al. 2016; Vismara et al. 2013). Despite these reviews indicating that practical limitations can be overcome, an ABA specific review by trained behaviour analysts will allow for a unique analysis of the methodology and outcomes reported in selected research.

Past reviews have also been limited to research utilising a parent training approach only (Meadan and Daczewitz 2015; Parsons et al. 2017). Meadan and Daczewitz (2015) selected six studies for their review, five of which were ABA-based interventions. Outcomes indicated that telehealth was an effective platform for parent training, increasing both parent’s knowledge and implementation skills. Parsons et al. (2017). ) reviewed research in using telehealth to conduct parent training in rural communities. The authors reviewed nine studies, all of which were behaviour analytic in nature, and concluded that, whilst parent training should be considered a crucial factor in intervention and has itself been identified as an EPB in the treatment of individuals with ASD (Wong et al. 2014), a more comprehensive review would provide a better insight into wider applications of telehealth across interventionists and beyond the home environment.

The most recent review focused on procedural fidelity and only selected studies that contained this measure (Neely et al. 2017). The authors concluded that all studies showed increases in interventionist implementation fidelity, demonstrating telehealth can be an effective platform. Admittedly, fidelity is an important factor for methodological rigor and high fidelity has been linked with optimal outcomes (Penn et al. 2007; Symes et al. 2006; Whiteford et al. 2012), however research focusing on outcomes for participants with ASD alone was overlooked.

Additionally, only one review to date investigated the quality of the included research (Parsons et al. 2017). Measures of research quality are essential as they allow for the assessment of research validity and indicate ability to minimise research errors and bias. Although Parsons et al. (2017) utilised a measure capable of simultaneously assessing the quality of multiple study designs, questions were more geared towards group designs and were somewhat subjective in nature (Kmet et al. 2004). Overall assignments of level of evidence were conducted using a separate grading system where single-subject research was automatically scored as the lowest level of evidence (National Health Medical Research Council 1999). ABA-based interventions are highly heterogeneous in nature; individuality of programming is prioritised over consistency of service between participants, allowing for optimal individual progress. Finding a quality rating capable of equitably comparing single subject and group research designs is paramount when assessing EBP. One such rating is the *Evaluative Method for Evaluating and Determining Evidence-Based Practices in Autism* (Reichow 2011; Reichow et al. 2008) which includes rubrics allowing to assess key indicators of quality in both single subject and group research designs. Scores on these rubrics can be combined to provide an overall level of EBP for the selected population.

The purpose of the current study is to systematically review and synthesise extant literature studying the effects of using telehealth to provide ABA-based provisions to individuals with a diagnosis of ASD. Main intervention components and outcomes will be extracted and combined to provide an overall picture of research aims, procedures, participants and effects. This will allow for analysis of success and identification of gaps in the literature. The *Evaluative Method for Evaluating and Determining Evidence-Based Practices in Autism* will be used to assess the methodological rigor of each study leading to an overall estimation of the status of telehealth as an EBP for the provision of ABA services to individuals with ASD.

## Method

Commencing in October 2017 with search dates ending in February 2018, we conducted a systematic search using four databases, ERIC, Medline, PsycInfo and Scopus. The review was conducted following the PRISMA checklist as a guide (Moher et al. 2009). Identified studies were screened by title and abstract, then merged with duplicates removed, followed by full text screening. Additionally, references of included studies and reviews on the topic were hand searched. A descriptive synthesis of eligible studies was then completed summarising the main objectives, variables and outcomes of each study. All selected studies were assessed for quality using standards set out by Reichow et al. (2008).

### Search Terms

**Fig. 1:**
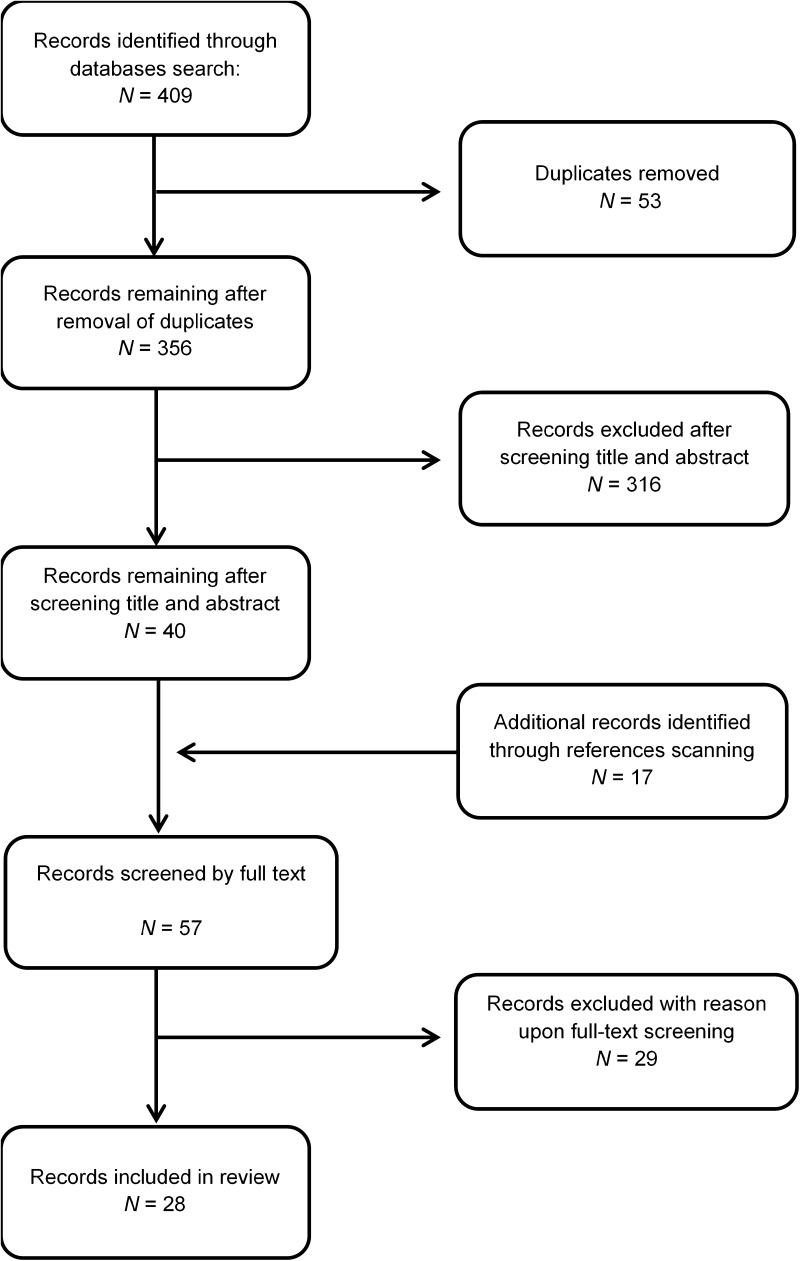
PRISMA diagram showing search strategy

Search terms included Autis* OR ASD OR Asperger OR PDD-NOS OR Developmental Disabil* AND Telehealth OR Telemedicine OR Teleconferencing OR Telecare OR Elearn* OR Distance Learn* (Fig. 1).

### Inclusion and Exclusion Criteria

For inclusion in the review, articles were examined against the following inclusion criteria: (1) peer review published journal article, (2) study involved telehealth technology in the provision of training, supervision or consultation to interventionists (parents or professionals) utilising behavioural principles in the treatment or education of at least one participant with a diagnosis of ASD, Asperger’s or PDD, (3) study was original empirical research with quantitative data and a primary analysis of the effect of an intervention conducted via telehealth and (4) was written in the English language.

Studies were excluded from the review if: (1) they did not include original research, (2) telehealth technology did not include a two-way communication system with a professional, (3) participants without a diagnosis of ASD or PDD were included and outcomes for the participants with ASD were not reported in isolation from other diagnoses.

### Data Extraction

Full texts of selected studies were reviewed and information was extracted (Table 1). The following measures were examined in order to develop an overview of the main intervention characteristics present in the research: (1) research design (2) participant characteristics (3) technology descriptions (4) dependent variables (5) intervention characteristics. Additionally, efficacy outcomes were reported to determine the overall success of the interventions. Research quality was scored following a quality assessment (Reichow et al. 2008) to provide an indication of research rigor. Additional information was collected, including measures of inter-observer agreement (IOA), generalisation and follow-up data.

**Table 1:** Descriptive information from selected studies

Study	Research design	Participants	Technology	Intervention category and aims	Dependent Variables	Outcomes	Quality
Barretto et al. (2006)	Single subject research; multi element design	Interventionists: two females, one teacher and one psychologist No ages reported Participants with ASD: one male Age: 5 years Diagnosis: ASD tool not reported	Iowa Communications Network (ICN) Camera that transmitted and recorded audio and visual information. Touch to speak Microphones Desktop with PowerPoint	Functional analysis Brief functional Analysis conducted via video conferencing. 5 min sessions. Free play, escape and alone. Interventionists received continuous live video conferenced coaching throughout	Interventionist variables: none reported ASD participant variables: child disruptive behaviour; screaming, noncompliance and property destruction	Positive Successful functional analysis identified escape function for behaviour	Weak
Barkaia et al. (2017)	Single subject research; multiple baseline design	Interventionists: three female therapists. Age: 24–32 (M = 27 years) Participants with ASD: three males Age: 4–6 years (*M* = 5 years 4 months) Diagnosis: ASD no diagnostic tool provided	Laptops and webcams used from centre Skype Viber/Mobile phone used for calls. videos sent to secured dropbox	Naturalistic teaching Didactic training: spoken and written instructions describing mand and echoic operants, practice exercise to discriminate. 1–2 h Coaching: video conferenced coached sessions focused on mand training and echoics, teaching contingencies of positive consequences, feedback and prompting opportunities. 10 min at end of coaching session recorded for analysis	Interventionist variables: fidelity/correct implementation of sequence and feedback Social validity questionnaire ASD participant: mands and echoic responses	Mixed All therapists increased correct commands from baseline Small increase in feedback for two participants larger increase for one Levels of fidelity were low < 60% Mixed increases in mands and echoics	Weak
Bearss et al. (2017)	Group research design; quasi experimental pre-post test with no comparison group	Interventionists: 13 parents Gender not specified Ages: mothers (*M* = 38.3), fathers (*M* = 39.8) Not clear which parent took part in trial Participants with ASD: 13, 9 males, 5 females Age: (*M* = 5.8 years) Diagnosis: ASD Diagnostic tool: ADOS	Equipment: Computers, scanners, projectors, cameras. No detailed specifics reported Videoconferencing Software: not reported	Behaviour support 6-month direct instruction parent training program designed to teach parents behaviour management strategies, conducted via videoconferencing. Trainers followed manualised training techniques including scripts and videos	Interventionist variables: treatment fidelity checklist Coaches ratings of success Parent treatment engagement scale Telehealth caregiver satisfaction survey Telehealth provider satisfaction survey Home Situations Questionnaire‑ASD Parent Satisfaction Questionnaire ASD participant variable Vineland Adaptive Behaviour Checklists Abhorrent behaviour checklist	Mixed High parent acceptability Trainers scored high levels of fidelity training via telehealth No significant differences in score of Vineland Significant improvements on the Social Withdrawal, Stereotypies Hyperactivity, and Inappropriate Speech subscales of the Abhorrent behaviour checklist	Weak
Benson et al. (2017)	Single subject research design; multi element/followed by an ABAB reversal design	Interventionists Families of ASD participant no information provided Participants with ASD: one male Age: 5 years Diagnosis: ASD diagnostic tool not reported	Dell™ Desktop computer external Logitech camera and Hangouts™ communications platform for video conferencing Debut video Captures software to record sessions	Functional Analysis and functional communication training Home based assessment and Intervention completed by parents with video conferenced coached training. Participants were live coached through the functional analysis This was followed by FCT Baseline consisted of participant being given access to maintaining reinforcement (tangible) after exhibiting SIB. The child was then prompted to use picture card as FCT to receive the item. Parents were live coached throughout	Interventionist variables Fidelity of parent procedures against task analysis of the assessment and training phases ASD participant variables SIB behaviour, face slapping Mands by touching or handing over communication card	Positive SIB decreased after intervention introduced and remerged upon return to baseline and mands increased and decreased on return to baseline	Weak
Gibson et al. (2010)	Single subject research design; ABAB reversal design	Interventionists two females, one pre-school teacher, one teaching assistant Ages not provided Participants with ASD one male Age: 4 years Diagnosis: ASD diagnostic tool not reported	Lap top with digital camera with inbuilt microphone. Skype Dell Latitude D820 notebook computer with a Microsoft Windows XP Logitech Quickcam	Functional communication training Witten direct instructions emailed, including a task analysis of intervention. Video consultation took place just before and after provided descriptive feedback Child taught to raise hand to access escape and toys during circle time	Interventionist variables: None reported ASD participant variables Elopement of ASD participant from assigned work area	Positive ABAB design showed clear return to baseline and improvement in treatment condition	Weak
Heitzman-Powell et al. (2014)	Group research design; quasi experimental design with pre-post test, no comparison group	Interventionists seven parents from four families, genders not provided Ages: 32–47 years. (*M* = 37.3 years) Participants with ASD: no details provided, referred as ‘children with autism’ in the abstract only	Online training delivered through an online learning management system Polycom® videoconferencing software	Comprehensive Training package on principles of ABA delivered through an online learning platform OASIS. Pre and post training knowledge assessments completed Video-conferenced coaching sessions with discussion of the topic and live coached sessions with participants own child	Interventionist variables Parent skill Assessment (Fidelity) completed before and after training. Scored from videotaped session Parent Knowledge Test Parent Satisfaction with the training ASD participant variables: none	Undetermined Parental gains on knowledge and fidelity were reported and appeared high, no statistical measures of this change were reported	Weak
Higgins et al. (2017)	Single subject research design; multiple baseline design	Interventionists: three female direct-care staff. All worked in EIBI for less than 6 months no experience with preference assessments Age: 21–24 years (*M* = 22.66 years) Participants with ASD: two males and one female Age: 4–5 years (*M* = 4 years) Diagnosis: ASD diagnostic tool not reported No pre-intervention assessment information provided	Adobe Connect 8 videoconferencing software Secure file encrypted file transferring software Laptop computer and Logitech Webcam Software v2.2	Preference assessments Participants taught to administer preference assessments via video conferencing Participants given access to written instructions prior to video conferencing Baseline taken on implementation of these instructions. First baseline was conducted using ASD participant, all subsequent training involved using a confederate actor. Training composed of multi-media presentation. Feedback on previous performance and on role play practice sessions. Followed by an assessment and two post training assessments, one with ASD participant	Interventionist participants: percentage of mastered components Social validity questionnaire ASD participant variables: none	Positive Increased fidelity for all participants in the implementation of skills, however only three data points were completed with Participants with ASD	Weak
Ingersoll and Berger (2015)	Group research design; randomly assigned comparison group	Interventionists: 27 parents, 96% female Age: not reported Participants with ASD: 27 participants 70% male Age: 23–73 months. (*M* = 3.7 years) Diagnosis: ASD diagnostic tools: ADOS, DSM-IV-TR Telehealth group *n* = 14 Comparison group *n* = 13 Pre intervention assessment: information on verbal mental age, non-verbal mental age and via Mullen scale of Early Learning	Home computers and webcams ImPACT online training website as per Ingersoll et al. (2016) Skype	Naturalistic teaching ImPACT online training designed to increase social communication Group 1: provided with access to website for 6 months Website consisted of 11 × 75 min lessons, video library, exercises and additional resources Group 2: in addition to the website participants were provided with 2 x weekly 30 min video conferencing session with trained coach One session introduced the topic the second provided live feedback 10-min recording of parent/child interaction during play or snack scored at baseline, post treatment and at 3 months follow up	Interventionist variables: depressive symptoms of parents Intervention knowledge Intervention fidelity Program engagement Program evaluation ASD participant variables: None	Positive Parent assist group more likely to engage with the website Parent engagement high for both groups Both groups significantly increased their intervention knowledge and fidelity, the video coached groups had significantly higher scores of fidelity post treatment	Weak
Ingersoll et al. (2016)	Group research design; randomly assigned comparison group Quasi experimental pre-post analysis for within group analysis	Interventionist participants: 27 parents Therapist Assisted Group (n = 14) Self-Directed group (n = 13) Gender and age not provided Participants with ASD: therapist assisted group (n = 14) 21% female and 79% male Age: (M = 41.57 months) Self-Directed group (n = 13) 13. 39% female and 61% male Age: (M = 46.08 months) Diagnosis: ASD or PDD-NOS Diagnostic tool: DSM-IV and ADOS Pre-assessment scores on the Mullen Scale of Early learning	ImPACT online training website with 12 self-directed lessons Video conferencing software not specified	Naturalistic teaching ImPACT online training designed to increase social communication. Comparison of two groups, one utilising video conferencing on top on online training Group 1: provided with access to website for 6 months Website consisted of 11 × 75 min lessons, video library, exercises and additional resources Group 2: in addition to the website participants were provided with 2 x weekly 30 min video conferencing session with trained coach	Interventionist variables: parent intervention fidelity Family Impact questionnaire (social validity) ASD participant variables: rate of use of individualised language targets, prompted and independent use of language scored MacArthur Bates Communication Development Inventory (Parent scored) Vineland Adaptive Behaviour scales (parent scored)	Positive Significant pre vs post-test increase in parent use of intervention scores of fidelity for both groups Therapist assisted group scored significantly higher at post intervention fidelity but this did not hold for 3 month follow up Increase in parental scores of self-efficacy and decrease in parental stress Significant language increases in child language use over time for language targets Small significant effect for group, therapist assisted group scored slightly better MCDI and vineland score significantly increased	Weak
Kuravackel et al. (2018)	Group research design; randomised control trial	Interventionist: 33 parents No gender or age reported Participants with ASD 33 participants, seven males and 26 females Face to face comparison group (*n* = 13) Age: 50–148 months (*M* = 104.62 months) Wait list comparison group (*n* = 10) Age: 39–153 months (*M* = 101.8 months) Telehealth group (*n* = 10) Age: 43–122 months (*M* = 82.3 months) Diagnostic tool: Modified Checklist for Autism in Toddlers (M-CHAT), Social Communication Questionnaire (SCQ), DSM-IV and ADOS	No information provided	Positive behaviour support/behaviour management Randomisation to face-to-face, telehealth or waitlist control. Parents provided with knowledge of ASD, and evidence based practices. Delivered via telehealth video conferencing in either a group or individualised format. A manualised copy of training procedures also provided	Interventionist variables: parental stress index Being a parent scale Consultation satisfaction questionnaire Group session rating scale Parent fidelity rating form ASD participant variables: EYEBERG child behaviour inventory Modified checklist for autism in toddlers Social Communication questionnaire	Mixed Significant difference in child problem behaviour scores No effects on parent outcomes	Weak
Lindgren et al. (2015)	Group research design; quasi-experimental design with comparison groups, no random assignment	Interventionist; 50 parent’s genders not provided Age: 23–51 years old Participants with ASD: Group 1 did not meet inclusion criteria for ASD diagnosis as outcomes were not reported separately Group 2: 20 participants, 19 males and one female Age: 29–80 months (*M* = 50.3 months) Group 3: 30 participants Age: 21–84 months (*M* = 50.3) Diagnosis: ASD, diagnostic tool not reported	Telehealth workstations equipped with Windows based PC, video monitor and headset Skype	Functional Analysis and Functional communication training Group 2 parents were coached via telehealth to conduct FAs and complete FCT interventions in clinic setting Group 3 conducted in home setting	Interventionist variables: acceptability ratings of the intervention ASD participant variables: % reduction in problem behaviour % increase in mands % increase in task completion	Positive Positive outcomes reported for reduction and increases in all DVs using within group analysis, however no statistical pre-post analysis completed No significant differences between home and centre based groups found after ANOVA	Weak
Machalicek et al. (2009a)	No experimental design	Interventionist: three graduate students in special education No age or gender provided Participants with ASD: three male participants Age: 34 months to 7 years (*M* = 4 years 11 months) Diagnosis: two ASD and one PDD-NOS diagnostic tool not reported	MacBookTM laptop iSightTM cameras iMacTM desktop iChatTM videoconferencing software JabraTM bluetoothwireless headset	Preference Assessment Trainee teachers taught paired choice preference assessments via video conferencing Participants provided with task analysis Trials were run through video conferencing with feedback and descriptive error correction	Interventionist variables: fidelity of procedures Social validity of the task ASD participant variables: frequency of items chosen	Undetermined No graphed data in report, teachers were reported to reach 100% fidelity within training period	Weak
Machalicek et al. (2010)	Single subject research design; multiple baseline design with additional embedded multi element components	Interventionists: six teachers all female Age: 22–32 (*M* = 27 years) Participants with ASD: six participants No genders provided Age: 4–10 years (*M* = 6 years) Diagnosis 5 ASD, 1 with autistic like behaviours Diagnostic tools not reported	MacBook laptop with additional speakers and microphone iSight camera iMac desktop used to film assessments iChat software used in video conferencing	Functional analysis During baseline participants were filmed completing an FA for each condition several times During video conference training participants were given performance feedback in real time and were error corrected by the supervisor	Interventionist Variables: teacher fidelity against a task analysis for each condition Supervisor/coach behaviour was assessed for fidelity against a pre-determined task list ASD participant variables: None	Positive All teachers demonstrated a large increase in the fidelity of treatment and reached fidelity criterion	Weak
Machalicek et al. (2016)	Single subject research design; multi-element/alternating treatments	Interventionists three parents, one male and two females. Ages not reported Participants with ASD: three participants Age: 8–16 years (*M* = 11 years) Diagnosis: ASD Diagnostic tool: DSM-V Pre-assessment info reported Childhood Autism Rating Scale (CARS)	2.4 Ghz/250 GB hard drive/SuperDrive MacBook™ Logitech QuickCam Pro 9000™ SuperDrive MacBook™ laptop computer with a built-in iSight™ web camera iChat™ videoconferencing software eCamm™ call recording software	Functional Analysis, FCT and Behaviour support strategies Phase one: initial teleconferenced Parents conducted an FA with telehealth support, prompting, error-correction and praise Phase two: treatment comparison parents were training in individualised support plans involving: antecedent strategies, FCT and DRA procedures Video modelling via telehealth was used to demonstrate each procedure	Interventionist variables: parental fidelity of FA procedures taken on 39%, 35% and 35% of FA sessions Parental procedural fidelity for 89%, 100% and 100% of FCT conditions Social Validity questionnaire ASD participant variables: occurrences of individualised target behaviour that challenges	Mixed Functional analysis indicted function for each participant, although un-labelled graphs make hard to determine Challenging behaviour was low for all conditions but there was no clear differentiation between conditions as the intervention was implemented	Weak
Machalicek et al. (2009b)	Single subject research design; multi element/alternating treatments	Interventionists: two graduate students No ages or gender provided Participants with ASD: two female participants Age: 11 and 7 years Diagnosis: ASD Diagnostic tool: childhood rating scale	2.0 Ghz Mac-Book™ laptop computers with Mac OS X operating system, 2 external iSight™ cameras, iChat™ videoconferencing software One laptop computer with iSight™ camera	Functional analysis Students were trained to conduct functional analysis via live video conferenced coaching	Interventionist variables: none ASD participant variable: individualised targeted behaviour that challenges	Positive Demonstrated clear function of behaviour for both participants	Weak
Meadan et al. (2016)	Single subject research design; multiple baseline design	Interventionists: three female parents Ages not provided Participants with ASD: three participants, two males and one female Age: 2–4 (*M* = 3) Diagnosis: ASD Diagnostic tool: preschool language score and Ages and Stages Questionnaire—social emotional	iPads provided to families used for video recording Skype Electronic materials shared via a secure online file sharing Box. Camtasia software to record sessions	Naturalistic teaching Internet-Based Parent-Implemented Communication Strategies (i-PiCS) First phase consisted of training delivered with a coach via skype lasting 45 min Second phase was direct coaching through the intervention which involved a 5-7-minute teaching session with the ASD participant Coaching took place two times per week and an annotated video feedback was provided every 4th coaching session	Interventionist variables: quality and rate with which the parent’s implemented the naturalistic teaching strategy (Fidelity) ASD participant variables: children’s social communication initiations and responses Additional fidelity testing on the coaching and training procedures to ascertain the fidelity of the coaching	Mixed Therapists demonstrated an increase in fidelity of implementation-on of strategies With very clear relationships demonstrated in 2 out of 3 days Children’s initiations increased over the intervention as did the percentage of successful communicate-on interactions for 2 out of 3 participants	Weak
Neely et al. (2016)	Single subject research design; multiple baseline design	Interventionists: three females currently working at University ABA clinic Age: 20–22 (*M* = 21 years, 4 months) Participants with ASD: three participants, two females and one male Diagnosis: two with ASD diagnosis 1 with PDD-NOS Diagnostic Tools: Participant A scores on ADOS, ASRS and Preschool Language Sale Student B only ASRS reported. Student C had no reported pre-intervention language outcomes	ipad mini used to record sessions Videos subsequently downloaded onto external hard-drive Vsee software used to conduct video conferenced using laptop 2.5-GHz ToshibaTM computer 2.4-GHz MacBookTM All interventionists used personal MacBooks Videoconferencing software, HIPPA-compliant	Naturalistic/incidental teaching Pre-intervention training; online module, self-evaluation and delayed feedback video modelling 5-min baseline videos of therapist working on target mand Followed by second video which was evaluated for fidelity independently by therapist and trainer and subsequently discussed through videoconferencing. Additional maintenance probes were carried out 2 and 4 months after fidelity reached	Interventionist variables: frequency of communication opportunities Percentage of incidental teaching steps performed correctly Scores on social validity Total duration of training ASD participant variable: child mands	Positive All therapists increased correct implementation of incidental teaching and reached fidelity All Participants with ASD increased manding	Adequate
Simacek et al. (2017)	Single subject research design; multi element alongside a multiple probe multiple baseline and ABAB reversal for first baseline	Interventionist Participants: parents of the ASD individuals no details were provided Participants with ASD: two females participants Age: 3.5 and 4 years Diagnosis: ASD Diagnostic tool not reported Functional assessment and structured descriptive assessment completed prior to intervention Scores of VABS, Vineland parent interview reported alongside previous and current services including EIBI for one participant	Dell OptiPlex 3010 Desktop with Dell 24in monitor, Logitech HD Pro Webcam C920, Logitech ClearChat Comfort/USB Headset H390 Google Hangout for video conferencing Debut screen recording software Parents used personal computer alongside as provided HD Pro Webcam C920	Functional analysis and functional communication training Coaches conducted a pre-intervention telephone call to carry out a functional assessment interview. Followed by a structured descriptive assessment to mimic the occurrences of targeted behaviours in the natural environment, no coaching occurred. Participants were instructed remotely on how to conduct the FA, with feedback being provided at the start of each session, throughout the session and via email FCT: baseline conducted where idiosyncratic behaviours were reinforced. Training was provided in the use of FCT using direct feedback and written instructions via email	Interventionist variables: fidelity to study procedures was measured for 20% of all outcomes using a task analysis of correct procedures Parent ratings of treatment acceptability ASD participant variables: individualised idiosyncratic behaviour (inappropriate ways to acquire reinforcement) Individualised AAC	Positive Functions of behaviour identified through FA. Both participant’s Idiosyncratic responses reduced to 0	Weak
Suess et al. (2014)	Single subject research design; multi-element and ABAB reversal designs	Interventionist: three parents gender not provided Age: (*M* = 37 years). No pre-intervention ABA experience provided Participants with ASD: three males Age: 29–39 months (*M* = 34 months) Diagnosis: PDD-NOS, diagnostic tool not reported. No pre-intervention assessment information provided	Laptop and Skype used for Video Conferencing, Debut videoconferencing software Electronic copy of training manual	Functional Assessment and Functional communication training Parents provided with two didactic training sessions via video conferencing. Covering FA, FCT and behaviour principles Parents subsequently coached via telehealth to conduct FA and FCT	Interventionist variables: fidelity to FCT task with adherence with personalised task analysis for sessions Type of error recorded Social validity questionnaire ASD participant variables: individualised challenging behaviour	Mixed FA successfully completed and identified a function for every participant Fidelity of FCT increased slightly for each participant and behaviour that challenges decreased, no functional relationship between coached and un-coached sessions and no baseline completed	Weak
Suess et al. (2016)	Single subject research design; multi element design with multiple baseline	Interventionists: parents of ASD individuals. No information provided Participants with ASD: five participants Three males and two females Age: 2.5–7.1 years (*M* = 5) Diagnosis: ASD, diagnostic tool not reported Pre-intervention descriptive functional assessment reported	AS per Wacker et al. (2013a) Skype	Functional analysis and Functional communication training Parents were trained via video conferencing to conduct FAs and subsequent FCT Initial 1 h meeting was conducted to discuss purpose and explain descriptive assessment FA was subsequently performed with ongoing instruction from the coach 3 × 15 min coached FCT sessions subsequently took place as per Wacker et al. (2013a)	Interventionist variables: none ASD participant variables: individually targeted behaviour that challenges Task completion mands	Positive Problem behaviour reduced for all participants Increased mands and task completion Statistical testing of effect size concluded that changed were significant	Weak
Vismara et al. (2013)	Single subject research design; multiple baseline design	Interventionists: eight parents of children involved, seven females and one male No ages reported Education, salary, employment status and marital status reported Participants with ASD: eight participants, no genders provided Age: 18–45 months (*M* = 27) Diagnosis: ASD Diagnostic tools, DSM-IV, ADOS used as a cut off for inclusion but not reported. Additional services received reported	Early Start Denver Model online training program Laptop for video calls, specific software details not provided	Naturalistic treatment program Video conferencing and use of a self- guided website on parent training in Early Start Denver Model (ESDM) Baseline was 10 min filming parent child interaction Weekly parent training sessions alongside video conferencing parent coaching sessions lasting 1.5 h	Interventionist variables: parent satisfaction, intervention skills, parent engagement styles maternal behaviour rating scale Parent website usage ASD individual outcomes: functional verbal utterances nonverbal joint attention Imitative play actions on objects and gestures	Mixed Parent engagement scores and fidelity scores increased from baseline for all participants ASD participant scores of verbal utterances and joint attention increased for some put not all of participants (multiple baseline not graphed)	Weak
Vismara et al. (2012)	Single subject research design; multiple baseline design	Interventionists: nine parents of children, seven females and two male No ages provided No pre-intervention experience reported Participants with ASD: nine participants, no genders provided Age: 16-38 months (*M* = 28.89) Diagnosis: six with ASD and three with PDD-NOS Diagnostic tools: ADOS Pre-assessment information provided including Mullen Scales of Early Learning and Vineland Adaptive Behaviour scales	Inter-based video conferencing software Computer, Laptops and webcams Training DVD in ESDM	Naturalistic treatment program Video conferenced coaching of ESDM 10-min video probes were completed at the start of each session, consisting of naturally occurring situations and evaluated skills using ESDM checklist Target behaviours were selected from these results Training DVD was provided 12 weekly video conferencing sessions teaching ESDM conducted	Interventionist variables: fidelity of implementation using the ESDM fidelity scale Maternal Behaviour Rating scale Feasibility and acceptability questionnaire ASD participant variables Child Social Communication Behaviours, prompted verbalisations, spontaneous verbalisations and spontaneous imitation MacArther Bates scores on vocabulary Vineland Adaptive Behaviour Rating scale	Positive Parent fidelity scores significantly improved over time	Weak
Vismara et al. (2016)	Group research design; randomised comparison group	Interventionists 24 parents Group 1: three males and 11 female Group 2: two male and eight females Ages not reported Participants with ASD: 24 participants Age: (*M* = 31.9 months) Diagnosis: ASD Diagnostic tool: ADOS Telehealth group: six males and four females Comparison group: 11 males and three females Additional services reported	Citrix program GoToMeeting® Parents accessed using home computer, webcam or tablet. Access to ESDM training website	Naturalistic treatment program Investigating parent’s use of ESDM Telehealth group Received access to ESDM online training ongoing weekly videoconferencing to coach them through ESDM procedures Comparison group Community treatment as usual group Received monthly videoconferencing not based upon the ESDM but discussing their current treatment, alongside access to the ESDM website Assessments were 5 min free-play time	Interventionist variables: P-ESDM fidelity checklist Parent website duration Parent satisfaction questionnaire ASD participant variables: spontaneous functional verbal utterances, Imitative functional play actions with or without objects Non-verbal joint attention	Mixed for ASD individual’s behaviour only significant behaviour difference between groups was imitation Significantly more parents in the telehealth group met fidelity after coaching	Weak
Vismara et al. (2009)	Group research design; non randomised comparison group	Interventionists: ten professionals recruited from selected centres, occupation, number of years’ experience working with individuals with ASD and previous training provided Age and gender not reposted Participants with ASD: 29 participants Age: 24–51 months. (*M* = 32) telehealth group and (*M* = 33) months for live group Diagnosis: ASD Diagnostic tool: ADOS	ESDM training DVD 2 day video conferenced training seminar (technology details not provided)	Naturalistic treatment program Phase 1 Baseline 10-min probe 2-day training conference 2 h training for each professional conducted via telehealth to discuss individual needs. 1-h phone-call follow up Phase 2 Professionals were given a parent training DVD and a 3-h didactic seminar on parent training, alongside a 2 h group supervision and 1 h telephone conference. Participants submitted a 1 h video of parent coaching session, self rated fidelity scores	Interventionist variables: interventionist and parent fidelity of implementation and interventionist satisfaction with the procedures ASD participant variables: frequency of child socio-communicative behaviours Imitative play actions on objects and gestures Observation ratings of child engagement	Positive Teaching via distance learning was as effective as teaching using live interaction No difference in therapist fidelity Attention and social initiation behaviours increased significantly from baseline	Weak
Wacker et al. (2013a)	Single subject research design: multi element with multiple baseline across participants for FCT component	Interventionists 18 parents, 16 females and two males Age: *M* = 33 years old No formal training in behavioural treatment Participants with ASD: 17 participants, gender not provided Age: 29–80 months Diagnosis: ASD or PDD-NOS diagnostic tool: DSM-IV, ADI and ADOS. Authors state that further diagnostic and demographic information can be found in subsequent publication	Sessions took place in regional telehealth clinics Windows based PC with teleconferencing software and basic webcam and microphone	FA and FCT training Parents attended a regional clinic where they were taught using video-conferencing to complete FA of problem behaviour and subsequent FCT FA procedures described in more detail in Wacker et al. (2013b) below FCT training via video conferencing in 1 h weekly session to coach through FCT procedures Five minute blocks were recorded throughout each session for scoring	Interventionist variables: acceptability of intervention ASD participant variables: % reduction in targeted challenging behaviour	Positive All participants had large reduction in targeted behaviour, six samples only graphed Parents rated the intervention highly	Weak
Wacker et al. (2013b) b Conduction Functional Analyse of Problem behaviour via telehealth	Single subject research design; multi element design	Interventionists: 20 parents of children, 19 females and one male Age: (*M* = 34 years) Participants with ASD: 20 participants, genders not provided Diagnosis: seven with ASD and 13 with PDD-NOS Diagnostic tools: DSM-IV, ADI and ADOS	Sony PCS-1600 videoconferencing system with PTZ camera Sony G520 video monitor Teleconsultation Dell Windows XP Logitech 600 Webcam Logitech G330 Emblaze-VCON vPoint HD software VideoLAN VLC media player Windows Movie Maker	Functional analysis Videoconferencing used to carry out training and completion of FA across four phases of training Initial training in phase 1 and 2 focused behaviour analytical procedures Parents interviewed about challenging behaviours and completed log and preference assessment Phase 4 Parents conducted an FA with support from the coach	Interventionist variables: Procedural integrity ASD participant variables: individualised target challenging behaviour, identified and operationally defined using pre-assessment interviews and logs	Positive Functions were successfully identified in 90% of cases, with the additional two cases not identifying a function due to low levels of behaviour that challenges	Weak
Wainer and Ingersoll (2015)	Single subject research design: multiple baseline design	Interventionist: five parents, all female Ages not reported Participants with ASD: five participants, gender not provided Age: 29–59 months (*M* = 42.2) Diagnosis: ASD Diagnostic tool not reported	Online Reciprocal Imitation Training (RIT) website Corresponding PDF manual Families own home computers and webcams Commercially available videoconferencing software, not named	Naturalistic teaching Hybrid approach of an online training program and video conferencing to investigate RIT 10-min baseline, filmed probes where parents interacted with their children in play Self -directed condition parents used online training program in RIT with four lessons, pre and post knowledge test video examples, active learning self-monitoring and homework 3 × 30 min coaching sessions involving 10 min probes a the start of session	Interventionist variables: RIT fidelity Parental engagement Parental knowledge Parental views on treatment acceptability ASD participant variables: child’s spontaneous imitation	Mixed Parental knowledge of RIT methods significantly increased Program fidelity increased from baseline for all participants, not significantly from self-directed to video conferencing Increased imitation for some children but not all, however significant relationship between parent fidelity and child imitation	Weak
Wilczynski et al. (2017)	No experimental design	Interventionists: one female special education teacher Age not provided ASD participant: one male participant Age: 5 years Diagnosis: ASD, diagnostic tool not reported	PC and webcam Autism Training Solutions web based training GoToMeeting video conferencing software	Comprehensive training Completion of online behaviour skills training; autism training solutions covering behaviour analytical principles Followed by video conferenced coaching session, training manual and provided feedback on pre-recorded videos	Interventionists variables: fidelity of implementation of each training component assessed Knowledge of interventions ASD participant behaviour: compliance with tasks completed, initial compliance and completion of task	Mixed Implementation of most training components increased after training Knowledge of key components increased ASD participant variable: small increase in initial compliance but completion of compliance was at ceiling levels pre-intervention	Weak

### Reliability of Search Procedures and Inter-coder Agreement (ICA)

To ensure internal validity within the review, the first and second authors independently assessed identified studies against inclusion and exclusion criteria. The two resulting lists of eligible studies were subsequently compared. ICA was calculated by dividing the total number of agreed eligible studies by the sum of all studies and multiplying by 100. A total number of two studies were disagreed upon leading to an ICA of 93%. Consensus was reached by discussing disagreements as a team leading to a final ICA of 100% on selected 28 studies. Likewise, ICA was calculated for the descriptive synthesis. The second coder scored 100% of selected studies across all extracted information. ICA was calculated by dividing the total number of agreed variables divided by total number of variables scored and multiplied by 100. There was a 92% agreement on coding. ICA was also calculated on the quality assessment at 96% following discussion on one disagreed study.

### Efficacy Outcomes

Intervention outcomes were rated for efficacy on an ordinal scale of ‘Positive’, ‘Mixed’ or ‘Negative’. Visual analysis of graphed data was used for studies employing single subject research designs, while statistical testing was employed to determine outcomes of group designs. Studies were considered ‘positive’ if improvements were made by all participants across all dependent variables. They were considered ‘Mixed’ if positive results were visible but did not apply to all dependent variables and ‘Negative’ if no improvements were made for any dependent variable.

### Quality Assessment

Studies included in the review were independently assessed for rigor by the first and second authors using the standards created by Reichow et al. (2008). Disagreements were discussed with the third author. This assessment used two rubrics to measure research quality, one for group research and one for single-subject research. Both consist of methodological elements deemed important for research rigor. Selected studies were assessed against the appropriate rubrics and an overall rigor rating was created using guidelines on how to synthesis rubrics ratings (Reichow et al. 2011, p. 30). This process evaluated study quality across two levels of methodological features: primary and secondary indicators. Primary indicators were considered vital components in research design in order to demonstrate validity. Secondary indicators were deemed as important but not vital components of research.

Primary indicators for group research included: participant characteristics, independent variables, comparison conditions, and dependent variable (Table 2). Primary indicators for single subject research included: participant characteristics, independent variables, baseline conditions, dependent variables, visual analysis and experimental control (Table 3). Secondary indicators for group research design included: (1) random assignment, (2), Inter-observer agreement (IOA), (3) blind raters, (4), fidelity, (5) attrition, (6) generalisation or maintenance, (7), effect size and (8) social validity. Secondary indicators for single subject research included: (1) inter-observer agreement (IOA), (2) kappa, (3), blind raters, (4) fidelity, (5) generalisation or maintenance and (6) social validity.

**Table 2:** Quality indicator assessment for group research designs

	Participants	IV	Com Con	DV	Link	Stat	RA	IOA	Blind Rater	Fidelity	Attrition	Gen/Main	ES	SV	Overall
Bearss et al. (2017)	H	H	U	H	H	H	N	N	N	E	E	N	E	E	Weak
Heitzman-Powell et al. (2014)	U	H	U	H	H	U	N	E	N	E	E	N	N	E	Weak
Ingersoll and Berger (2015)	U	H	H	H	H	H	E	N	N	N	N	N	N	E	Weak
Ingersoll et al. (2016)	U	H	H	H	H	H	E	E	N	E	N	E	N	E	Weak
Kuravackel et al. (2018)	U	H	H	H	H	H	E	N	N	N	N	N	E	E	Weak
Lindgren et al. (2015)	U	H	A	H	H	H	N	E	N	N	E	N	N	E	Weak
Vismara et al. (2016)	U	H	H	H	H	H	E	N	E	E	E	E	N	E	Weak
Vismara et al. (2009)	U	H	A	H	H	U	N	E	E	E	E	N	N	E	Weak

*IV* independent variable, *RA* random assignment, *ES* effect size, *U* unacceptable, *E* evidence of Com, *Con* comparison condition, *IOA* interobserver agreement, *SV* social validity, *A* acceptable, *N* no evidence of *DV* dependent variable, *BR* blind rater, *H* high, *Stat* statistical testing, *Gen*/*Main* generalisation/maintenance

**Table 3:** Quality indicator assessment for single subject research designs

Study Name	Participant characteristics	IV	Baseline	DV	Visual analysis	Experimental control	IOA	Kappa	Fidelity	Blind raters	Generalisation or maint	Social validity	Overall
Barretto et al. (2006)	U	H	U	H	H	H	E	N	N	N	N	E	Weak
Barkaia et al. (2017)	A	H	A	H	A	A	E	N	E	N	N	E	Weak
Benson et al. (2017)	U	H	U	H	A	A	E	E	E	N	N	E	Weak
Gibson et al. (2010)	U	H	H	H	H	H	E	N	E	N	N	E	Weak
Higgins et al. (2017)	A	H	U	H	U	U	E	N	E	N	E	E	Weak
Machalicek et al. (2009b)	U	H	U	H	H	H	E	N	N	N	N	E	Weak
Machalicek et al. (2010)	A	H	U	H	A	U	E	N	N	N	E	E	Weak
Machalicek et al. (2016)	U	H	U	H	U	U	E	N	E	N	N	E	Weak
Machalicek et al. (2009a)	U	H	U	H	U	U	E	N	E	N	N	E	Weak
Meadan et al. (2016)	U	H	A	H	A	A	E	N	E	E	E	E	Weak
Neely et al. (2016)	H	H	A	H	H	H	E	N	E	N	E	E	Adequate
Simacek et al. (2017)	U	H	A	H	H	H	E	N	E	N	E	E	Weak
Suess et al. (2014)	U	H	U	H	U	U	E	N	E	N	N	E	Weak
Suess et al. (2016)	U	H	U	H	A	A	E	N	N	N	N	E	Weak
Vismara et al. (2012)	U	H	A	H	A	H	E	E	E	E	E	E	Weak
Vismara et al. (2013)	U	H	A	H	U	U	E	E	E	E	E	E	Weak
Wacker et al. (2013a)	H	H	U	H	A	A	E	N	E	N	N	E	Weak
Wacker et al. (2013b)	U	H	A	H	A	A	E	N	N	N	N	E	Weak
Wainer and Ingersoll (2015)	U	H	H	H	A	A	E	N	E	N	E	E	Weak
Wilczynski et al. (2017)	U	H	U	H	U	U	N	N	E	N	N	E	Weak

*IV* independent variable, *U* unacceptable, *E* evidence of *DV* dependent variable, *A* acceptable, *N* no evidence of *IOA* interobserver agreement, *H* high, *Gen*/*Main* generalisation/maintenance

A score sheet created by the authors was used to score each of the identified variables against the operational definitions set out in Reichow et al. (2011). Each primary variable was scored as ‘High (H)’, ‘Acceptable (A)’ or ‘Unacceptable (U)’. Secondary indicators were scored as ‘evidence (E)’ or ‘no evidence (N)’. Each score was then combined to give an overall quality rating for each study of ‘strong’, ‘adequate’ or ‘weak’. To receive an overall ‘strong’ rating, studies must have received a high rating for all primary indicators and meet four of the secondary indicators. An overall ‘adequate’ rating was awarded to a study that received a high rating for four primary indicators and meet two secondary indicators with no unacceptable ratings in primary indicators. A study received an overall ‘weak’ rating if it was awarded less than four high ratings in primary indicators and less than two secondary indicators. Final ratings of each study were amalgamated and assessed using the formula set out by Reichow (2011, p. 31), which allowed for an overall rating of EPB to be assigned to the field.

## Results

According to the inclusion criteria, a total of 28 studies were deemed eligible and were incorporated in the descriptive synthesis with key data extracted and coded (Table 1). Each study was subsequently examined to determine its quality according to the indicators identified by Reichow et al. (2008). In the following sections, a summary of coded variables and quality measures is presented.

### Study Design

All but two of the selected studies (Machalicek et al. 2009a; Wilczynski et al. 2017) used an experimental research design, with the majority (64%; *n* = 18) employing a single subject research design and fewer employing a group design (28%; *n* = 8). Of the single subject research multiple baseline designs were utilised in 36% of studies (*n* = 10), as were multielement designs. Reversal designs were rarely used in the research (14%; *n* = 4).

### Participants

#### Interventionist Participant

A total number of 293 interventionists were included in the 28 studies. Of these, 68 were placed in a comparison group and did not undertake any training via telehealth, leaving 225 interventionists in experimental groups across all studies. Of these, 86% (*n* = 194) were parents, 9% (*n* = 21) were direct front line staff, including ABA therapists, graduates working in university clinics and other associated professionals working in the field, and 4% (*n* = 10) were teachers.

Age and gender for the interventionists were reported rarely with only 25% (*n* = 7) of studies reporting both characteristics (Bearss et al. 2017; Barkaia et al. 2017; Higgins et al. 2017; Neely et al. 2016; Machalicek et al. 2010; Wacker et al. 2013a, b). Another 25% (*n* = 7) of studies reported neither age nor gender (Benson et al. 2017; Kuravackel et al. 2018; Machalicek et al. 2009a, b; Simacek et al. 2017; Suess et al. 2016; Vismara et al. 2009). Gender alone was reported in 39% (*n* = 11) of studies (Barretto et al. 2006; Gibson et al. 2010; Ingersoll et al. 2016; Ingersoll and Berger 2015; Machalicek et al. 2016; Meadan et al. 2016; Vismara et al. 2012, 2013, 2016; Wainer and Ingersoll 2015; Wilczynski et al. 2017). Age alone was reported in 11% (*n* = 3) of studies (Suess et al. 2014; Lindgren et al. 2015; Heitzman-Powell et al. 2014).

In studies where interventionist demographics were reported, a total of 97 females and 14 males took part in the research. The average age of interventionists was 31.7. Few participants had previous experience implementing behavioural analytic procedures (14%; *n* = 16).

#### Participants with ASD

A total of 307 participants with ASD took part in the 28 studies. Of this, 76 were allocated to a control or comparison group and did not receive telehealth interventions, leaving 231 participants in telehealth experimental groups. Of these 57%, (*n* = 231) took part in group research, while 42% (*n* = 96) were included in single subject research designs and 2% (*n* = 4) were included in studies that did not operate an experimental research design.

Age and gender of participants with ASD were reported more frequently than for interventionists, with both variables being reported in 75% (*n* = 21) of studies (Barretto et al. 2006; Barkaia et al. 2017; Bearss et al. 2017; Benson et al. 2017; Gibson et al. 2010; Higgins et al. 2017; Ingersoll and Berger 2015; Kuravackel et al. 2018; Lindgren et al. 2015; Machalicek et al. 2009a, b, 2010, 2016; Meadan et al. 2016; Neely et al. 2016; Simacek et al. 2017; Suess et al. 2014, 2016; Vismara et al. 2016; Wilczynski et al. 2017). Only 3% (*n* = 1) of studies did not report any information about the age or gender of participants with ASD (Heitzman-Powell et al. 2014). Participants’ age was stated in 21% (*n* = 6) of studies in which gender was omitted (Vismara et al. 2009, 2012, 2013; Wacker et al. 2013a, b; Wainer and Ingersoll 2015), while no studies reported gender in isolation.

Diagnostic tools used to provide the participants with their diagnoses of ASD or PDD-NOS were reported sporadically throughout the literature. Of the demographics reported for participants with ASD, 93 were male and 61 were female. The average age from each study where age was reported was 4.73 years with the range being 1.75–16 years’ old.

### Intervention Characteristics

#### Categorisation of Intervention Aims

**Fig. 2:**
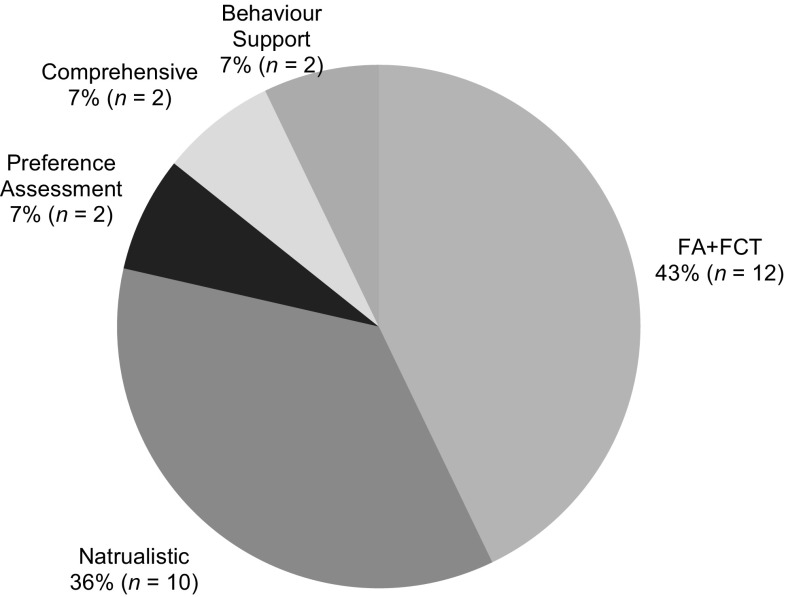
Proportion of studies completing research in each intervention category

Studies included in this review were organised according to the intervention techniques used (Fig. 2). The largest category was functional analysis (FA) and subsequent functional communication training (FCT), with 43% (*n* = 12) of studies investigating these topics (Barretto et al. 2006; Benson et al. 2017; Gibson et al. 2010; Lindgren et al. 2015; Machalicek et al. 2009b, 2010, 2016; Simacek et al. 2017; Suess et al. 2014, 2016; Wacker et al. 2013a, b). Techniques derived from naturalistic and incidental teaching were employed in 36% (*n* = 10) of studies (Barkaia et al. 2017; Ingersoll et al. 2016; Ingersoll and Berger 2015; Meadan et al. 2016; Neely et al. 2016; Vismara et al. 2009, 2012, 2016; Wainer and Ingersoll 2015). Behaviour support strategies, including positive behaviour support were investigated in 7% (*n* = 2) of studies (Bearss et al. 2017; Kuravackel et al. 2018). Training participants to conduct preference assessments was examined in 7% (*n* = 2) of studies (Higgins et al. 2017; Machalicek et al. 2009a). The final two studies focused on comprehensive training packages designed to provide participants with an overview of behaviour analytic principles (Heitzman-Powell et al. 2014; Wilczynski et al. 2017).

### Training Characteristics

When examining eligible studies, it was apparent that several commonalities occurred across training techniques and platforms used. These included technology-based training and video-conference sessions that provided coaching. Coaching was defined as providing individualised training and feedback regarding the implementation of the intervention of choice; this could be conducted via telephone, email or video-conferencing. Feedback on performance was delivered live or retroactively using recorded footage. Coaching was used as a standalone training strategy 25% (*n* = 7) of studies (Barretto et al. 2006; Benson et al. 2017; Lindgren et al. 2015; Machalicek et al. 2009b, 2016; Simacek et al. 2017; Suess et al. 2016).

Technology-based training involved the utilisation of technology to provide interventionists with the theoretical background knowledge of procedural techniques in preparation of intervention commencement. Twenty studies included a technology based training components were included in 71% (*n* = 20) of studies. Written instructions, websites including interactive modules, a training DVD, manuals or with video-conferenced didactic training were utilised to provide this training. Equipment included laptops, cameras, scanners and commercially available videoconferencing software. A hybrid approach including coaching and technology-based training was undertaken in 75% (*n* = 21) of studies (Barkaia et al. 2017; Bearss et al. 2017; Gibson et al. 2010; Heitzman-Powell et al. 2014; Higgins et al. 2017; Ingersoll and Berger 2015; Ingersoll et al. 2016; Kuravackel et al. 2018; Machalicek et al. 2009a, 2010; Meadan et al. 2016; Neely et al. 2016; Suess et al. 2014; Vismara et al. 2009, 2012, 2013, 2016; Wacker et al. 2013a, b; Wainer and Ingersoll 2015; Wilczynski et al. 2017).

### Dependent Variables

Results indicate that 69% (*n* = 19) of studies measured variables for both interventionists and participants with ASD, 11% (*n* = 3) of studies measured interventionist behaviour alone. All studies that measured interventionist behaviours included a measure of procedural fidelity via task analysis, a fidelity checklist or video-recorded probes. Knowledge tests were used in 14% (*n* = 4) of studies and these scores were compared to pre-intervention scores to determine the effect of the intervention (Heitzman-Powell et al. 2014; Ingersoll and Berger 2015; Wainer and Ingersoll 2015; Wilczynski et al. 2017).

Child behaviour was measured alone in 21% (*n* = 6) of studies and 68% (*n* = 19) of studies measured participant with ASD variables alongside interventionist variables. Data collection for child behaviours was conducted via video-recorded probes or questionnaires and standardised tests. Of these, 50% (*n* = 14) of studies measured individualised problem behaviour as part of an FA to assess function or teach replacement behaviour (e.g. FCT). Examples of the individuals’ challenging behaviour include elopement (Gibson et al. 2010), self-injurious behaviour and screaming (Benson et al. 2017), noncompliance and property destruction (Barretto et al. 2006). From the remaining studies, 43% (*n* = 12) investigated increases in social-communication responses (Barkaia et al. 2017; Benson et al. 2017; Ingersoll et al. 2016; Lindgren et al. 2015; Meadan et al. 2016; Neely et al. 2016; Simacek et al. 2017; Suess et al. 2016; Vismara et al. 2009, 2012, 2013, 2016) All studies attempted to teach children to request using either an alternative communication system (e.g., touching or handing over a communication card to access a tangible; Benson et al. 2017) or vocalisations (e.g., providing echoic prompts in contrived communication opportunities to increase child vocal requesting; Barkaia et al. 2017). Other facets of social communication measured including joint-attention and initiation of communication (e.g., Neely et al. 2016).

The final dependent variable measured in the research was imitation skills. This was included in 18% of studies (*n* = 5) (Vismara et al. 2009, 2012, 2013, 2016; Wainer and Ingersoll 2015) all five studies trained parents in naturalistic teaching techniques and attempted to increase children’s imitation skills in play based or fun situations.

### Efficacy Outcomes

**Fig. 3:**
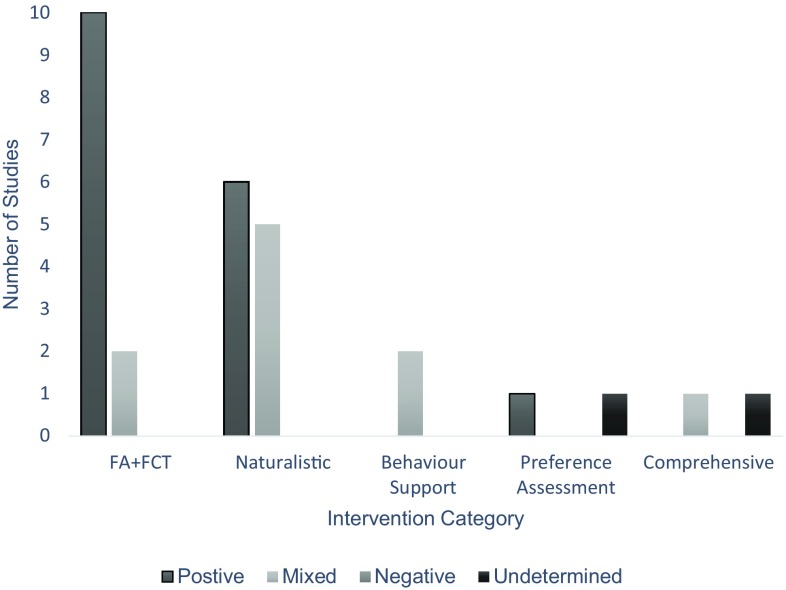
Efficacy outcomes of each intervention category

Results of efficacy (Fig. 3) show that 61% (*n* = 17) of studies were rated as ‘positive’ in which improvements were achieved by all participants across all dependent variables (Barretto et al. 2006; Benson et al. 2017; Gibson et al. 2010; Higgins et al. 2017; Ingersoll and Berger 2015; Ingersoll et al. 2016; Lindgren et al. 2015; Machalicek et al. 2010, 2009b; Neely et al. 2016; Simacek et al. 2017; Suess et al. 2016; Vismara et al. 2009, 2012, 2016; Wacker et al. 2013a, b). A closer examination reveals that 36% (*n* = 10) of studies employed FA + FCT procedures (Barretto et al. 2006; Benson et al. 2017; Gibson et al. 2010; Lindgren et al. 2015; Machalicek et al. 2009b, 2010; Simacek et al. 2017; Suess et al. 2016; Wacker et al. 2013a, b) demonstrating a clear behaviour function for each participant and showing decreases in challenging behaviour following FCT; in terms of procedural fidelity, this was established for all interventionists. Of studies focusing on naturalistic teaching, 35% (*n* = 6) were also scored as positive (Ingersoll et al. 2016; Ingersoll and Berger 2015; Neely et al. 2016; Vismara et al. 2009, 2012, 2016), achieving an increase in interventionist knowledge or fidelity alongside improvements in child social communicative behaviour or imitation responses. The final positively scored study included a preference assessment (Higgins et al. 2017) and indicated a positive relationship between telehealth training and the correct implementation of preference assessment procedures.

Overall, 32% (*n* = 9) of studies received a ‘mixed’ efficacy rating (Barkaia et al. 2017; Bearss et al. 2017; Machalicek et al. 2016; Meadan et al. 2016; Suess et al. 2014; Vismara et al. 2013, 2016; Wainer and Ingersoll 2015; Wilczynski et al. 2017). For example, 44% (*n* = 4) of these studies found improvements in interventionist treatment fidelity across all participants but failed to increase scores of social communication or imitation behaviours consistently across participants (Meadan et al. 2016; Wainer and Ingersoll 2015; Vismara et al. 2013). None of the 28 studies included in this review received a ‘negative’ rating.

### Quality Assessment

**Fig. 4:**
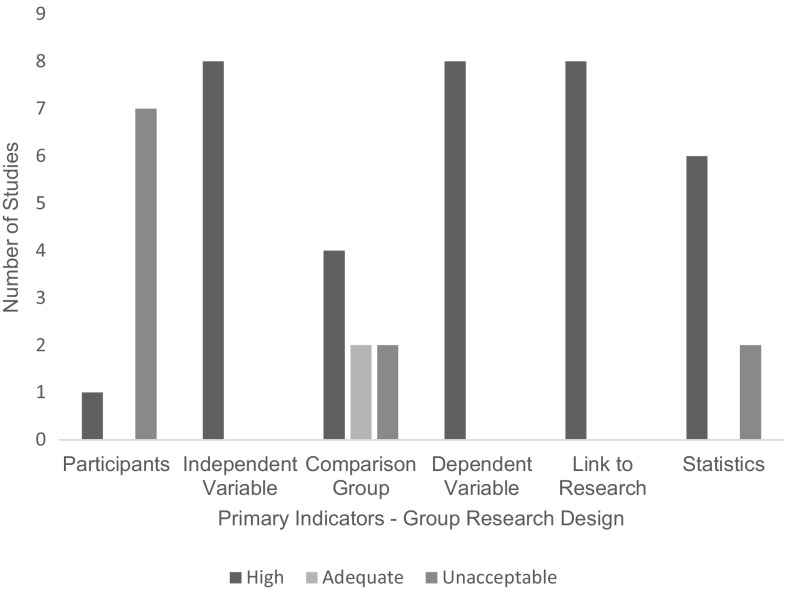
Number of studies scoring on each primary indicator—group research design

**Fig. 5:**
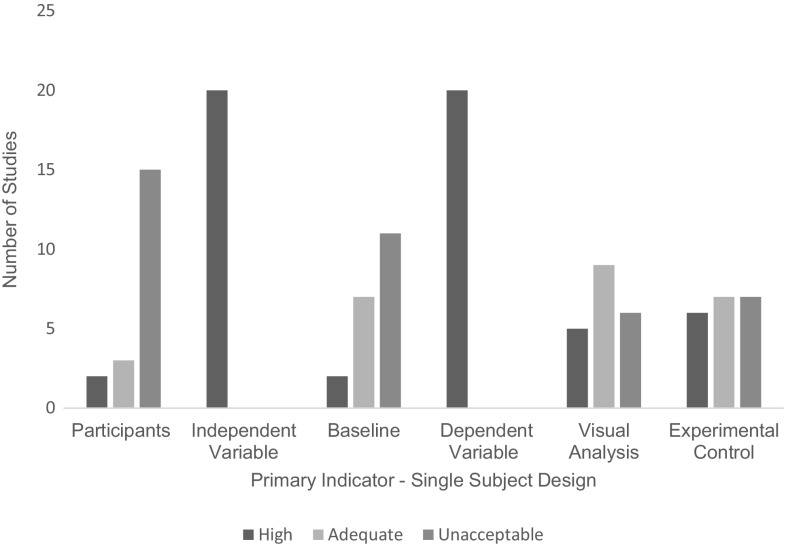
Number of studies scoring on each primary indicator—single subject research design

Of the eight studies employing a group research design, all (*n* = 8) received an overall weak quality rating (Fig. 4). Of the 71% (*n* = 20) of studies employing a single subject research design (Fig. 5), 5% (*n* = 1) received an overall adequate quality rating; the remaining 95% (*n* = 19) received an overall weak quality rating. An overall rating for each study can be found alongside individual indicator ratings in Tables 2 and 3.

The research scored highly on several primary indicators, including both the dependent and independent variables, which were described thoroughly with replicable precision in 100% of single subject and group research. In group research links to past research were successfully made in 100% of studies meaning they provided strong links between the original research questions and the analysis of the data established in their studies. Comparison groups, when present, were described with high replicable detail in 50% (*n* = 4) of studies and with some details emitted in 25% (*n* = 2). The remaining studies operated a within group design and did not include a comparison group to describe. Statistical analyses were also completed to a high standard in 75% (*n* = 6) of studies (Bearss et al. 2017; Ingersoll and Berger 2015; Ingersoll et al. 2016; Kuravackel et al. 2018; Lindgren et al. 2015; Vismara et al. 2016) as all contained sample sizes > 10 participants per group and conducted appropriate statistical analysis across dependent variables.

However, high scores were not evident across all indicators. In single subject research design studies only 10% (*n* = 2) for studies were rated highly for baseline (Gibson et al. 2010; Wainer and Ingersoll 2015), meaning they had three measurable data points, were described in replicable details, appeared to be stable and did not include any trends, including counter therapeutic trends. An additional 35% (*n* = 7) were rated as ‘Acceptable’ as more than 50% but less than 100% of baselines in the study met the above criteria (Barkaia et al. 2017; Meadan et al. 2016; Neely et al. 2016; Simacek et al. 2017; Vismara et al. 2012, 2013; Wacker et al. 2013b). 55% of studies (*n* = 11) were rated as having unacceptable baseline conditions. Despite conditions being accurately described and for the most part containing three data points, many failed to show stable levels or trends.

For both single subject and group research to be rated ‘High’ for participant characteristics studies needed to report information on participant age, gender, diagnostic instrument, interventionist characteristics and scores on standardised tests if applicable. As many of the studies included measures of both interventionist and participants with ASD behaviour both were considered as participants for the purpose of this review. Overall only 11% (*n* = 3) of the studies were rated highly. This means they reported information on participant age, gender, diagnostic instrument, interventionist characteristics and scores on standardised tests if applicable. An ‘Acceptable’ rating was awarded to 15% (*n* = 3) of studies, as they provided demographics on all participant’s age and gender, and provided standardised test scores. The remaining 79% (*n* = 22) of studies all met the ‘Unacceptable’ rating, where age and gender for both the individual with ASD and the interventionists was not reported consistently.

The overall quality ratings for each study were combined to allow for an overall rating of EPB to be given to the field. Using the formula suggested by Reichow et al. (2011) the amount of ‘high’ and ‘adequate’ studies were combined to provide an overall score for the field, which due to methodological omissions discussed was designated as ‘Not an Evidence Based Practice’.

## Discussion

The purpose of this review was to (a) identify and categorise key intervention properties and procedures used in research using telehealth to provide behaviour analytic provisions to individuals with ASD, (b) to assess the overall outcomes of selected studies both in the success of the training procedures and the outcomes for individuals with ASD, and (c) to examine the quality of selected research. It is the aim of the review that it can be used to guide future research and practice by identifying successful procedures and highlighting methodological flaws.

Findings from the synthesis of 28 studies suggested that telehealth can be an acceptable platform for behaviour analytic interventions and assessments. A total of 293 interventionists were trained across studies providing intervention to 307 individuals with ASD. Outcomes indicate positive gains across participants with 100% of studies (*n* = 28) studies reporting improvements in at least one dependent variable and 61% (*n* = 17) of studies reporting favourable outcomes across all dependent variables. None of the 28 studies met sufficient quality indicators to be determined as of ‘high’ quality and only one study was determined as being of ‘adequate’ quality. The remaining 96% (*n* = 27) of studies were rated as ‘weak’. Due to what we consider are minor methodological flaws, an overall rating of ‘not an evidence-based practice’ was assigned to telehealth as a means of providing ABA-based interventions. Although positive outcomes were achieved for involved participants, there is a demand for further high quality research that can adhere to a rigorous methodological structure. Each of the research aims will now be discussed in more detail.

The first aim of this review was to synthesise and categorise intervention aims and procedures. A variety of ABA-based procedures were targeted within the literature: FA and FCT, naturalistic teaching, preference assessments, behaviour support and comprehensive programs. The numbers of studies in each category were not evenly distributed, most studies investigated FA and FCT procedures (43%; *n* = 12) or naturalistic teaching (39%; *n* = 11). Research on using telehealth to conduct comprehensive treatment is lacking. Only 7% (*n* = 2) of selected studies were categorised as ‘comprehensive’ (Heitzman-Powell et al. 2014; Wilczynski et al. 2017), but both had methodological downfalls. Using telehealth to oversee comprehensive packages is a vital area for future research with potentially significant practical implications. Two other key ABA-based teaching strategies were overlooked in the research: Discrete Trial Training (DTT) and functional/daily living skills training. DTT is highly repetitious and includes very structured arrangements of consequences and antecedents. It has been shown to successfully teach skills to children with ASD across developmental domains such as, communication, imitation and self-care (Sheinkopf and Siegel 1998). DTT implementation requires technical knowledge and success is linked to good training procedures (Symes et al. 2006). On the other hand, DTT has been criticised as teaching skills that present a lack of generality into typical, ecologically relevant settings. Further research is needed to determine its suitability through a telehealth model.

Conversely, functional/daily living skills may be ideally suited. Strategies of task analysis and chaining are used to systematically teach ‘chains’ of behaviours found in everyday tasks, such as brushing teeth or preparing a snack. These methods have a strong empirical base, have been recognised as evidence-based practice (Wong et al. 2014) and have been demonstrated using a parent training model (Kroeger and Sorensen 2010), highlighting their generality and social validity. It is therefore relatively surprising that research into using telehealth to conduct this training is not forthcoming and future research in this direction is needed.

A total of 71% (*n* = 20) of studies included an initial training period. Initial training has been recognised as a key component in achieving procedural fidelity in behaviour analytic practice (Denne et al. 2015; Symes et al. 2006). Researchers have translated proven face–face training techniques onto a telehealth platform, such as modelling of procedures, practice using role play and tests of knowledge (Fetherstone and Sturmey 2014; Miltenberger 2004). All 28 studies incorporated a live coached component, in addition to initial training or as a stand-alone training procedure. Once again, proven training techniques were utilised, such as modelling appropriate behaviour, error correction procedures and performance feedback. Due to using a combination of training variables it was not possible to isolate the most successful training components. Future researchers may consider a component analysis to assess the best type of training and avoid unnecessarily waste of resources.

Information regarding the type of technology was limited within the research with only a small number of studies providing extensive information on this aspect. Video conferencing software used to conduct didactic training and video coaching was often free to access and readily available, e.g., Skype, Viber or iChat. Hardware such as personal computers, web cameras or tablets were often reported as family’s own or pre-existing in the intervention centre. Initial training was conducted via a website in eight studies, where website training was utilised this was often already available from previous research, such as the Early Start Denver Model training website (e.g. Vismara et al. 2016).

The review synthesised participant information. By determining who can be trained to carry out interventions and who is likely to benefit from them, the scope of the telehealth model can be revealed. A total of 225 interventionist participants took part. Participants were employed in a number of different sectors: health, education, research and social care or were family members, primarily parents of the participants with ASD. This synthesis demonstrates the capacity of telehealth to train and supervise a multi-disciplinary team, highlighting the potential of the telehealth model amongst the reality of current ASD services in the UK, where interdisciplinary teams working together to plan provision is common practice (Department of Education, Department of Health 2015; Department of Education Northern Ireland 2005).

The largest proportion of studies (64%; *n* = 18) used parent training to support the provision of home-based intervention or assessments. The National Research Council (NRC) dictates that parent involvement is a fundamental component of effective ASD intervention (NRC 2001). Parent training and subsequent intervention implementation has been identified as evidence-based practice (EBP), as long as treatment fidelity can be achieved (Wong et al. 2014). The outcomes of this review indicate that fidelity can be achieved using a telehealth model and combination of telehealth and parent training has a promising future.

The age of participants with ASD ranged from 1.75–16 years, with the majority of participants being under 6 years old. Current research trends indicate that young age is a crucial predictor of success in behavioural interventions (Perry et al. 2011). The UK government initiatives have highlighted early intervention as a crucial focus for future research (National Institute for Health Research 2017). The application of telehealth with this age cohort is very promising, enabling parents to receive training prior to the commencement of educational services could be key in future service models. Despite the promising prospects of early intervention, the research is limited to this narrow age range. Prior research shows that ABA-based intervention can have great success with both a teenage and adult age cohort (Bennet and Dukes 2013; Koegel et al. 2014; Santiago et al. 2016). Future research might focus on the application of these interventions with an older age group allowing for a greater concept of the scope of telehealth.

The second aim of the review was to assess the outcomes of the research in order to determine if the interventions were successful. An overview of selected research indicates at least some favourable outcomes in all 28 studies. Outcomes were measured for both interventionists and participants with ASD and varied depending on intervention aims and category.

A total of 75% (*n* = 21) of studies collected data on at least one measure of interventionist behaviour. All but one collected data on procedural fidelity. Measures of fidelity have been shown to correlate with best outcomes for child participants (Penn et al. 2007; Symes et al. 2006; Whiteford et al. 2012). All 21 studies showed improvement in procedural fidelity for all participants involved. However, one study (Barkaia et al. 2017) reported improved measures of fidelity but still achieved a relatively low fidelity level of around 60% which would not be considered widely acceptable. Several other studies reported the need to perform ‘top up’ training in order for agents to reach pre-set fidelity criteria. Despite these few discrepancies, the literature does demonstrate the capabilities of telehealth and goes some way to answering the pertinent question of whether behaviour analytic provisions can be delivered via a telehealth with appropriate levels of fidelity to ensure best outcomes. Additional studies should also focus on collecting fidelity on the coach’s procedures whilst training interventionists, this data was collected by a small number of studies (e.g. Neely et al. 2016) and allows for a tertiary level of data collection to ensure a greater level of procedural integrity across all levels of the research.

Data on outcomes of participants with ASD were collected in 85% (*n* = 24) of studies. Measures differed based upon the intervention category but as a whole improvement was less consistent than measures of fidelity for interventionists. Measures of challenging behaviour were collected in 50% (*n* = 14) of studies, social-communication responses were collected in 46% (*n* = 13) of studies and motor imitation was measured in 17% (*n* = 5). Overall, there was improvement in target behaviour across studies, although several studies observed little or no improvements in some but not all participant outcomes (e.g. Barkaia et al. 2017; Machalicek et al. 2016; Meadan et al. 2016; Suess et al. 2014; Vismara et al. 2013; Wainer and Ingersoll 2015). A greater proportion of naturalistic interventions were rated as mixed when compared to FA + FCT studies (45% vs. 17%). For example, Barkaia et al. (2017) found clear gains in mand and echoic behaviour in one out of three participants. Whether this was a result of individual differences or a failure of the telehealth model remains to be seen, although as mentioned earlier this study reported low levels of therapist fidelity. In a similar study Neely et al. (2016) were able to achieve a high rating of fidelity across therapists and increased manding for all participants with ASD. This was the only study to be rated as of ‘adequate’ quality. Individual differences in outcome success have often perplexed ABA researchers. On-going research aims to identify factors which may predict success (Mudford et al. 2009; Perry et al. 2011; Whiteford et al. 2012). This area is somehow explored in several of the group design studies included in this review (e.g. Ingersoll et al. 2016; Vismara et al. 2009, 2016) but further research is needed in this area.

The final aim of the present systematic review was to rate the methodological quality of the existing body of evidence. All 28 eligible studies were assessed against the research quality indicators developed by Reichow et al. (2008). Overall ratings of quality were low and telehealth-based applications of ABA are currently deemed to have a status of ‘Not an EBP’.

In the single subject research design studies, more than half of the studies (39%; *n* = 11) were rated as having unacceptable baseline conditions. Despite conditions being accurately described and for the most part containing three data points, many failed to show stable levels or trends. This is perhaps a result of the applied nature of the research in which it is not always practical or even possible to wait for stable trends of baseline responding. Future research should aim to establish a stable baseline level, so that it can provide a stronger demonstration of a functional relation between the intervention and behaviour change and higher levels of internal validity. Pre-planning resources so as to enable extension of baseline if stable levels of behaviour are not achieved in the first three data points should be conducted.

Experimental control was demonstrated to an acceptable level in 13 studies; similarly, 14 studies were adequate for visual analysis. Research deemed unacceptable failed to demonstrate experimental control as visual analysis showed an unacceptable level of overlapping data or absence of three instances of experimental control. Future studies should ensure that these essential aspects are present by increasing participants or replications.

The participant demographic indicator achieved the lowest score, with 79% (*n* = 22) of studies being deemed unacceptable. Only two studies were rated highly in this indicator (Neely et al. 2016; Wacker et al. 2013a) and age and gender of interventionists was rarely reported. Detailed demographics allow for the generalisation and replication of findings; by omitting this information, researchers undermine external validity. It is vital that future research reports detailed demographics of both interventionists and participants, including level of education and experience and diagnosis and pre-intervention assessment respectively. Future researchers should consider providing all participant demographics for both the participants with ASD and the interventionists. This should include age and gender, pre assessment tools and previous experience. High methodological rigor in this area is demonstrated by the three studies within this review that scored highly on the participant indicator (Bearss et al. 2017; Neely et al. 2016; Wacker et al. 2013a).

Omission of age or gender was quite often the limiting factor restricting quality ratings across studies amongst both single subject research design and group research design. If participant details had been reported even to an adequate standard, three additional group design studies and two additional single subject studies would have gained an overall quality rating of ‘adequate’ (Gibson et al. 2010; Ingersoll et al. 2016; Kuravackel et al. 2018; Lindgren et al. 2015; Simacek et al. 2017) and one group design study would have achieved a ‘strong’ rating (Vismara et al. 2016). With this in mind the formula for determining EBP can be reapplied (Reichow 2011, p. 34). The research would now be provided with a higher score (*z* = 79), which would in turn translate to a rating of an ‘Established EBP’. This is a vast difference from the original score. It could be argued that this is a weakness of the quality assessment, which allows relatively small omissions to have such a large effect on ratings; however, as discussed previously, participant information is a vital component in the establishment of external validity therefore collecting and reporting key information is of crucial importance. Interestingly, no group research design studies were rewarded an acceptable rating for the participant indicator, whilst 15% (*n* = 3) of single subject research design studies scored an acceptable rating. This may allow for conclusions that single subject research design holds a higher standard of research rigor in comparison to group research designs. It is also important to note that only 36% (*n* = 10) of the reviewed 28 studies included maintenance or follow-up probes. Again, of these studies just 25% (*n* = 2) of group research designs showed evidence of maintenance probes whilst 50% (*n* = 10) of single subject research designs reported this variable. This adds to the conclusion that a single subject research design may hold itself above group research design in research quality and the individualised nature and repeated measurements are more suited to the heterogeneity of behaviour analytic interventions. Of all 28 studies, just 30% (*n* = 3) had promising follow-up data. This should be a prioritised area of focus for future research.

Current research surrounding telehealth interventions as a means to train interventionists, although still limited, is progressing. The limitations we have identified will aid in the development of methodologically strong studies The use of telehealth is not aimed to replacing face-to-face behavioural interventions but to complement or boost their results. Future reviews are needed to assess the outcomes of a combination of telehealth and face–face models of delivery, which were excluded from this current review. Additionally, all eligible studies were conducted in the United States, therefore future research should focus on the feasibility and cost effectiveness of similar provisions in different countries in which cultural differences might impact on existing models.

In sum, this systematic review suggests that training interventionists to implement behaviour analytic provisions for children with ASD via telehealth is feasible and effective. Small improvements in research rigor could lead to this delivery model being deemed an EBP. Future researchers should familiarise themselves with quality indicators to ensure methodologically robust research is conducted.
